# Gut Microbiota Composition in Maintenance Hemodialysis Patients: Associations with Sex, Age, and Body Composition

**DOI:** 10.3390/nu18111682

**Published:** 2026-05-25

**Authors:** Katarzyna Bąk, Michał Kowalski, Kamila Marszalek, Patrycja Olszewska, Andrzej Ossowski, Bartłomiej Grygorcewicz, Aleksandra Cader-Ptak, Leszek Domański, Violetta Dziedziejko, Ewa Kwiatkowska

**Affiliations:** 1 Clinical Department of Nephrology, Transplantology and Internal Medicine, Pomeranian Medical University, 70-001 Szczecin, Poland; katarzyna.bak@pum.edu.pl (K.B.); aleksandra.cader@pum.edu.pl (A.C.-P.); leszek.domanski@pum.edu.pl (L.D.); 2 Department of Genomics and Forensic Genetics, Pomeranian Medical University, 70-001 Szczecin, Poland; michal.kowalski@pum.edu.pl (M.K.); patrycja.olszewska@pum.edu.pl (P.O.); andrzej.ossowski@pum.edu.pl (A.O.); bartlomiej.grygorcewicz@pum.edu.pl (B.G.); 3 Department of Biochemistry and Medical Chemistry, Pomeranian Medical University, 70-001 Szczecin, Poland; violetta.dziedziejko@pum.edu.pl

**Keywords:** gut microbiota, hemodialysis, chronic kidney disease, body composition, gut–kidney axis

## Abstract

Background/Objectives: Patients receiving maintenance hemodialysis (HD) commonly exhibit chronic low-grade inflammation, nutritional disturbances, altered body composition, and metabolic imbalance. Gut dysbiosis may contribute to these abnormalities through the gut–kidney axis; however, the relationship between the gut microbiota composition and host phenotype in HD patients remains incompletely characterized. This study aimed to characterize the gut microbiota composition in maintenance HD patients and assess its cross-sectional associations with demographic, inflammatory, nutritional, dialysis-related, and bioimpedance-derived body composition parameters. Methods: This single-center cross-sectional study included 96 patients with end-stage kidney disease undergoing maintenance HD. The primary objective was to characterize the gut microbiota composition in maintenance HD patients. Secondary objectives were to assess cross-sectional associations with demographic factors (sex, age) and bioimpedance-derived body composition (specifically VAT). Clinical and laboratory data, inflammatory markers, nutritional indicators, malnutrition–inflammation score (MIS), dialysis-related variables, and bioimpedance-derived body composition parameters were collected. Stool samples were analyzed using full-length 16S rRNA sequencing. The gut microbiota composition was assessed using taxonomic profiling, alpha-diversity and beta-diversity analyses, subgroup comparisons, and exploratory distance-based analyses. Associations were interpreted within a descriptive and hypothesis-generating framework. Results: The gut microbiota composition showed marked inter-individual heterogeneity at the genus level, with dominant taxa including *Blautia*, *Faecalibacterium*, *Streptococcus*, *Gemmiger*, *Ruminococcus*, *Escherichia-Shigella*, and *Enterococcus*. Chao1 richness was higher in men than in women. Shannon entropy and Chao1 richness were positively associated with age and visceral adipose tissue (VAT), while Faith’s phylogenetic diversity increased with age. In contrast, the Gini index was negatively associated with age and VAT, indicating a more even microbial community structure in older individuals and in those with higher visceral adiposity. Beta-diversity analyses suggested modest differences in microbial community structure according to sex and selected body composition-related categories, particularly in sex-stratified analyses. Exploratory distance-based analysis showed a modest association between overall microbiota dissimilarity and host phenotype dissimilarity, although this finding was limited by reduced sample overlap. Conclusions: The gut microbiota composition in maintenance HD patients was highly heterogeneous and showed cross-sectional associations, mainly with sex, age, visceral adiposity, and broader host phenotype. These findings suggest that microbiota variation in HD reflects multidimensional demographic, inflammatory, nutritional, metabolic, and body composition-related factors rather than a single clinical determinant. Larger longitudinal studies integrating standardized dietary, medication, metabolic, and clinical outcome data are needed to determine the prognostic relevance of these microbiota patterns.

## 1. Introduction

Chronic kidney disease (CKD) represents a growing global health burden and a major contributor to cardiovascular morbidity and mortality, affecting approximately 4.5 million individuals in Poland alone. Patients who progress to stage 5 CKD require kidney replacement therapy, most commonly maintenance intermittent hemodialysis (HD). In Poland, nearly 20,000 patients undergo chronic HD treatment. Despite advances in dialysis technology and supportive care, mortality among hemodialysis patients remains alarmingly high, reaching up to 30% within two years of follow-up [1].

Malnutrition and chronic inflammation are highly prevalent in this population and are strongly associated with adverse outcomes. Protein–energy wasting (PEW) affects up to 75–90% of patients receiving dialysis, while a persistent low-grade inflammatory state is observed in nearly all individuals undergoing HD. The interrelationship between malnutrition, inflammation, and cardiovascular disease is conceptualized as malnutrition–inflammation–atherosclerosis (MIA) syndrome—a complex and often fatal condition in end-stage renal disease (ESRD), in which these interdependent factors coexist and mutually amplify one another, leading to accelerated vascular disease and excess mortality. MIA is frequently described as a vicious cycle, where each component exacerbates the others.

Malnutrition (M), typically defined as protein–energy wasting, is characterized by hypoalbuminemia, reduced body mass, and loss of skeletal muscle mass. Inflammation (I) is reflected by elevated circulating markers such as C-reactive protein (CRP), interleukin-6 (IL-6), and tumor necrosis factor-α (TNF-α). Atherosclerosis (A) represents accelerated vascular injury and remains the leading cause of death in dialysis patients [2].

The pathogenesis of MIA syndrome is multifactorial. Chronic inflammation in HD patients is driven by the retention of proinflammatory uremic toxins—including indoxyl sulfate and p-cresyl sulfate—which promote sustained immune activation and endothelial dysfunction [3,4,5]. Additional contributors include vascular access-related infections and complications [6], as well as dialysis-related inflammatory stimuli triggered by blood–membrane interactions, dialysate contaminants, and nutrient losses during treatment [7,8,9]. Comorbidities, such as diabetes, obesity, pre-existing atherosclerosis, chronic infections, and psychosocial factors including depression, further potentiate systemic inflammation and worsen metabolic reserve [8,9].

Dietary restrictions imposed on dialysis patients—particularly limitations of fluid, sodium, potassium, and phosphorus intake—often lead to insufficient caloric and protein consumption. Depression, which is common in this population, may additionally suppress appetite. Moreover, each hemodialysis session is associated with amino acid and iron losses [10], and the procedure itself promotes a catabolic state, partly mediated by inflammatory signaling [11]. Chronic inflammation and malnutrition together accelerate atherosclerosis, creating a self-reinforcing feedback loop that worsens clinical prognosis.

In recent years, growing attention has been directed toward the gut–kidney axis as a central mechanism linking CKD to systemic inflammation and cardiovascular disease. CKD and ESRD are consistently associated with gut dysbiosis characterized by reduced microbial diversity, depletion of beneficial short-chain fatty acid (SCFA)-producing bacteria, and enrichment of taxa capable of generating precursors of uremic toxins. Colonic microbial metabolism substantially contributes to circulating levels of indoxyl sulfate, p-cresyl sulfate, and trimethylamine-*N*-oxide (TMAO), metabolites that accumulate as renal function declines and are associated with endothelial dysfunction, oxidative stress, and increased cardiovascular risk [12,13,14].

Mechanistically, CKD-associated dysbiosis is accompanied by increased intestinal permeability (“leaky gut”), facilitating translocation of endotoxins and other microbial products into the circulation, thereby amplifying systemic immune activation. Concurrently, reduced SCFA production may impair intestinal barrier integrity and anti-inflammatory immune pathways, including regulatory T-cell responses, further reinforcing chronic inflammation and metabolic disturbances relevant to protein–energy wasting and cardiovascular injury [12].

Emerging multi-omics and translational evidence supports the concept of a “toxic microbiome” in CKD, in which progressive enrichment of uremic toxin-producing species and depletion of protective taxa accompany disease advancement. Experimental studies have demonstrated that fecal microbiota transplantation from CKD patients increases circulating uremic toxin levels and exacerbates kidney fibrosis in animal models, suggesting that gut microbial alterations may actively contribute to CKD progression rather than merely reflect it [15,16,17].

Collectively, these findings provide a biologically plausible link between gut dysbiosis, systemic inflammation, metabolic imbalance, and accelerated atherosclerosis—key components of MIA syndrome. However, despite increasing recognition of the gut–kidney axis in CKD, the relationship between the gut microbiota composition and the clinical manifestations of MIA in maintenance hemodialysis patients remains insufficiently characterized [18].

Therefore, the primary aim of this study was to characterize the gut microbiota composition in patients undergoing maintenance hemodialysis and to assess its cross-sectional associations with demographic characteristics, inflammatory and nutritional markers, dialysis-related variables, and bioimpedance-derived body composition parameters. Particular emphasis is placed on sex-, age-, and body composition-related microbiota variations, in line with the descriptive and hypothesis-generating nature of the study.

## 2. Materials and Methods

### 2.1. Study Design and Ethical Approval

This study was approved by the Bioethics Committee of the Pomeranian Medical University in Szczecin (approval number KB-006/17/2025). All participants provided written informed consent before inclusion.

A total of 96 patients with stage 5 chronic kidney disease undergoing maintenance intermittent hemodialysis (HD) at the Independent Public Clinical Hospital No. 2 of the Pomeranian Medical University in Szczecin were enrolled. The study consisted of four main components:

Blood sampling for the assessment of inflammatory and nutritional markers;

Stool collection for gut microbiota analysis;

Body composition analysis;

Nutritional assessment using the malnutrition–inflammation score (MIS).

**Figure 1:**
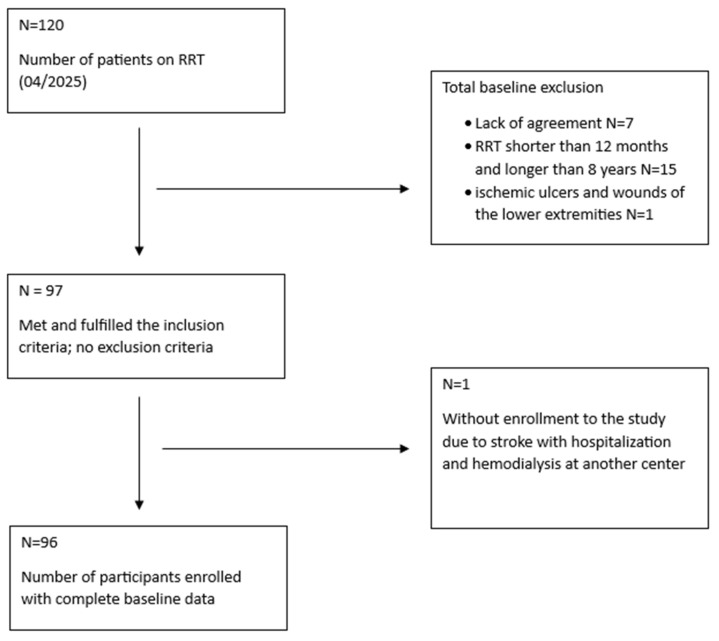
Participant recruitment flowchart.

The baseline characteristics of the study population are presented in Table 1, and the participant recruitment flowchart is shown in Figure 1.

### 2.2. Study Endpoints

The primary aim of this study was to characterize the gut microbiota composition in patients undergoing maintenance hemodialysis (HD) and identify the dominant microbial taxa and inter-individual heterogeneity within this population.

The secondary aims included:

Assessment of sex-specific differences in gut microbiota alpha- and beta-diversity;

Evaluation of the associations between age and microbiota diversity metrics;

Analysis of the relationship between bioimpedance-derived body composition parameters (with a focus on visceral adipose tissue, VAT) and microbiota structure;

Exploratory assessment of the associations between the microbiota and clinical/inflammatory markers, including vascular access type and neutrophil count.

### 2.3. Body Composition Analysis

Body composition was assessed using a validated medical bioimpedance analyzer (seca mBCA 525, SECA GmbH, Hamburg, Germany), according to the manufacturer’s instructions [19]. Body weight and height were measured manually prior to bioimpedance analysis. Measurements were performed after a hemodialysis session to minimize the effect of fluid overload. The examination was rapid and non-invasive, based on an 8-point bioelectrical impedance measurement. The applied current was 100 μA. Due to safety considerations, patients with implanted cardiac devices were excluded. Body composition and anthropometric parameters, including BMI, FFM, FFMI, FM, FMI, TBW, phase angle, and VAT, were included to characterize nutritional status, adiposity, hydration, and cellular integrity; definitions and interpretations of these variables are provided in Table 2.

Body composition assessment was performed using a rapid and non-invasive 8-point bioelectrical impedance analysis conducted on the patient’s body surface. The electrical current applied during the measurement was 100 μA; therefore, individuals with implanted cardiac electronic devices were excluded for safety reasons. To minimize the influence of fluid overload, all measurements were performed immediately after a hemodialysis session.

Participants were categorized according to body mass index (BMI) into standard clinical groups: starvation (<16.0 kg/m^2^), emaciation (16.0–16.99 kg/m^2^), underweight (17.0–18.49 kg/m^2^), normal weight (18.5–24.99 kg/m^2^), overweight (25.0–29.99 kg/m^2^), obesity class I (30.0–34.99 kg/m^2^), obesity class II (35.0–39.99 kg/m^2^), and obesity class III (>40.0 kg/m^2^).

The SECA body composition analyzer automatically classified results according to reference ranges adjusted for age, sex, ethnicity, and BMI. Fat mass index (FMI) was categorized as low, normal, elevated, or high, while visceral adipose tissue (VAT) was classified as decreased, normal, or increased. Phase angle values were interpreted as reduced (<5°), normal (5–7°), or elevated (>7°), reflecting cellular integrity and membrane function.

Fat-free mass index (FFMI) was analyzed separately for women and men using sex-specific reference values [20]. In women, values below 14 kg/m^2^ were considered below average, 14–18 kg/m^2^ as normal, and above 18 kg/m^2^ as high. In men, values below 18 kg/m^2^ were classified as below average, 18–25 kg/m^2^ as normal, and above 25 kg/m^2^ as high muscle mass.

### 2.4. Inflammatory and Nutritional Markers

Blood samples were collected prior to the hemodialysis session during routine monthly laboratory assessments. Samples were obtained from the arteriovenous fistula or dialysis catheter after withdrawal of the locking anticoagulant solution. Blood was drawn into 4 mL EDTA tubes and centrifuged at 4000 rpm for 10 min, and plasma was stored at −80 °C until further analysis.

Inflammatory status was assessed by measuring high-sensitivity C-reactive protein (hsCRP), interleukin-6 (IL-6), interleukin-1β (IL-1β), and tumor necrosis factor-α (TNF-α).

Plasma concentrations of classical inflammatory markers were measured using commercially available enzyme-linked immunosorbent assay (ELISA, Helsinki, Finland) kits. Quantikine HS ELISA kits (Bio-Techne R&D Systems, Abingdon, UK) were used for the determination of hs IL-6, hs IL-1β, and hs TNF-α. Hs CRP concentrations were determined using ELISA kits from DRG Instruments GmbH (Marburg, Germany). All analyses were performed according to the manufacturers’ protocols. Absorbance measurements were carried out using a BioTek Cytation 5 Cell Imaging Multimode Reader (Winooski, VT, USA).

The concentrations of nutritional status markers (albumin and transferrin) were obtained from routine biochemical tests performed in the Central Laboratory of the University Clinical Hospital No. 2, Pomeranian Medical University in Szczecin.

Nutritional and inflammatory burden was further assessed using the malnutrition–inflammation score (MIS) as originally described by Kalantar-Zadeh et al. [21]. The MIS incorporates elements of medical history (including weight changes, appetite, gastrointestinal symptoms, and functional capacity), physical examination findings related to fat and muscle wasting, body mass index (BMI), and laboratory parameters such as albumin and transferrin levels. A total MIS was calculated for each participant.

### 2.5. Clinical and Laboratory Data

Demographic and clinical data were collected for each participant, including age, sex, dialysis vintage, type of vascular access, dialysis adequacy (Kt/V), primary cause of CKD, comorbidities (including cardiovascular disease, diabetes, malignancy, chronic obstructive pulmonary disease, and autoimmune disorders), and current medication use.

Routine laboratory parameters analyzed included hemoglobin, ferritin, transferrin, albumin, calcium, phosphate, and parathyroid hormone (PTH).

No participants met the KDIGO criteria for erythropoiesis-stimulating agent (ESA) resistance, defined as an erythropoietin (EPO) dose exceeding 300 IU/kg/week subcutaneously or 450 IU/kg/week intravenously without adequate hemoglobin response. Therefore, the erythropoietin resistance index (ERI) was used as a continuous measure of ESA responsiveness.

ERI was calculated as the mean weekly EPO dose per kilogram of body weight divided by the mean hemoglobin concentration (g/dL), based on values obtained over the preceding three months. As no universally accepted ERI cut-off exists to define ESA hyporesponsiveness, patients were analyzed according to median values and tertiles.

### 2.6. Bioinformatics

The bioinformatic methodology employed in this study encompasses a comprehensive and reproducible analysis of full-length 16S rRNA marker sequences, and it was fine-tuned to the sequencing parameters.

### 2.7. Preprocessing

#### Basecalling

The initial preprocessing phase took place simultaneously with sequencing on an Oxford Nanopore Technologies Promethion 2 Integrated. The sequencer itself allows for robust utilization of a dual flow cell configuration. The *real-time basecalling* was performed on the HAC (High ACcuracy) *Dorado* model (5.2.0, 400 bps). The choice of HAC models for *real-time basecalling* was dictated by the hardware bottleneck of the NVIDIA A100GPU (graphics processing unit) onboard the Promethion 2 Integrated, which cannot handle *real-time basecalling* on a dual flow cell setup with SUP (SUPer accuracy) basecalling models due to insufficient processing capacity that introduces lags to the sequencing summary [https://github.com/Kirk3gaard/2025-Crowdsource-GPU-basecalling-stats] (accessed on 20 February 2026). Despite the induced processing delays, the implementation of real-time basecalling was methodologically necessary. This approach provided the crucial advantage of continuous monitoring of barcode abundance and library uniformity throughout the sequencing Such real-time evaluation was strictly required to determine the optimal operational termination point of the sequencing run, allowing the sequencing to be strategically stopped, when the evaluated samples successfully reached the lowest acceptable passed reads threshold (70,000 reads).

Following initial basecalling, the raw POD5 files were basecalled with use of the standardized *wf-basecalling* pipeline (version 1.5.7) from the EPI2ME framework (version 5.3.1), utilizing the SUP basecalling model (5.2.0). The pipeline was configured to trim all the adapters and barcodes from the SQK-16S114-24 barcoding kit Oxford Nanopore Technologies (ONT), Oxford, UK, present in the data.

### 2.8. Processing

#### Curation of Metadata

The collected metadata were stored in the form of an XLSX sheet and curated using the Python libraries *Pandas* (2.2.2) and *Numpy* (1.26.4). The columns regarding anthropometric indices were processed from continuous to factorial, using the following constraints:

1. Body mass index (BMI): categorized into starvation (<16.0 kg/m^2^), emaciation (16.0–16.99 kg/m^2^), underweight (17.0–18.49 kg/m^2^), normal weight (18.5–24.99 kg/m^2^), overweight (25.0–29.99 kg/m^2^), obesity class I (30.0–34.99 kg/m^2^), obesity class II (35.0–39.99 kg/m^2^), and obesity class III (>40.0 kg/m^2^).
2. Fat mass index (FMI): divided into four levels—low, normal, elevated, and high—as specified in the database.
3. Visceral adipose tissue (VAT): classified into three categories: normal, elevated, and high.
4. Phase angle: grouped into three ranges: decreased (<5°), normal (5–7°), and increased (>7°).
5. Fat-free mass index (FFMI): segmented by gender with specific cutoff points.
  ○ For females: below average (<14 kg/m^2^), good muscle mass (14–18 kg/m^2^), and high muscle mass (>18 kg/m^2^).
  ○ For males: below average (<18 kg/m^2^), average (18–25 kg/m^2^), and high (>25 kg/m^2^).

The continuous variables presenting given metrics were also utilized in this study at stages where continuous variables are required (e.g., alpha-diversity correlation).

Analysis with standardized wf-16s pipeline from EPI2ME framework.

To validate the sequencing and explore whether samples make biological sense, the wf-16s pipeline (1.6.0) was run, configured to perform taxonomic classification to the SILVA 138.1 database (EPI2ME had no default option to use the SILVA 138.2 database) with use of the *minimap2* aligner [22]. The reports were thoroughly examined to search for outliers. The *wf-16s* pipeline does not take into account phylogenetic analysis and statistical testing in its alpha-diversity and beta-diversity analyses; thus, subsequent employment of a different approach was necessary.

### 2.9. Quality Control and Filtering

Quality control and filtering for the data coming from ONT long-read sequencing requires dedicated tools such as the *NanoPlot* (1.46.2) and *NanoFilt* (2.8.0). These are used to present the quality control results and to filter sequences according to specified parameters [23].

The initial quality control was carried out on unfiltered data to check the read length and read quality distributions across all samples. Each sample was investigated individually, and a bulk report was created with MultiQC (1.33) [24]. The minimum read length threshold was set to 800 base pairs and the maximum read length threshold to 2200 base pairs to remove clear artifacts from the generated data. However, these settings were found to be arbitrary, as the range of the length was conservatively estimated at 1500 base pairs, meaning that the sequencing did not produce any artifacts. The quality threshold was set to a PHRED score of 12, as the amount of data below that threshold made about 5% of generated reads, and the mean PHRED score of raw data equaled 24.3, with 114,400 reads per sample on average.

After quality filtering with NanoFilt, the second round of quality control with NanoPlot and MultiQC took place, providing sequences with a mean PHRED score of 24.3, with 113,500 reads per sample on average.

### 2.10. Preparation for Analysis with Qiime2

As the quality of data coming from the SUP model basecalling does not require any read correction, and denoising methods such as DADA2 or Deblur are incompatible with them, the preprocessed BAM files were converted to FASTA as sequences of high fidelity and certainty. To be able to fully utilize the *Qiime2* framework, the ONT-sequenced data coming from the complementary strand must be rewritten to match the original template strand with “vsearch orient” and using the SILVA 138.2 SSU Ref NR99 database. The FASTA files records headers were then signed with the corresponding sample ID, and the sample manifest in TSV format was prepared to serve as an input for the Qiime2 framework.

### 2.11. Downstream Analysis

The downstream analysis was performed fully using Qiime2 2026.1 (rachis) framework with Python API. (Python 3.14).

### 2.12. OTU Construction and Sequence Processing with Vsearch

The OTU construction and sequence clustering was performed using the “vsearch” plugin [25] and the dereplication action “dereplicate-sequences”, with chimera filtering carried out using “uchime-ref” action, and clustered to the reference database using the “cluster_sequences_closed_ref” action with the identity threshold set to 97% and abundance threshold set to a minimum of 1. Only the sequences clustered to the reference were passed onto the next steps of the analysis.

A closed-reference 97% OTU clustering strategy was selected as a conservative approach for full-length 16S rRNA Oxford Nanopore sequencing data. Because ONT long-read amplicon data may contain platform-specific errors and because the primary aim of this study was ecological comparison between clinically defined host phenotypes rather than discovery of novel bacterial lineages, sequences were clustered against the curated SILVA 138.2 reference database. This strategy provided a standardized reference-anchored feature table suitable for downstream diversity, phylogenetic, and taxonomic analyses. Only sequences matching the reference database were retained for further analysis, thereby reducing the influence of potentially spurious, non-target, or poorly classified sequences. We acknowledge that this approach may exclude novel, rare, or insufficiently represented taxa and therefore limits discovery-oriented interpretation at finer taxonomic resolution [26].

### 2.13. Construction of Phylogenetic Tree

The phylogenetic tree for calculation of phylogenetic alpha-diversity and beta-diversity metrics was constructed with the “phylogeny” plugin, using the “align-to-tree-mafft-fasttree” pipeline on the sequences clustered to the reference at the OTU construction step [27,28].

### 2.14. Exploratory Data Analysis

To investigate hidden data properties, such as confounding factors, the exploratory data analysis was performed on the whole dataset without any stratification.

### 2.15. Diversity Analysis

Diversity analysis was performed using the “diversity” plugin, including alpha rarefaction curves to determine the minimum acceptable sampling depth for phylogenetic analysis; beta rarefaction curves to observe how rarefaction affects the beta-diversity metrics; core phylogenetic and non-phylogenetic alpha-diversity and beta-diversity metrics such as Faith phylogenetic diversity [29], unweighted UniFrac Striped UniFrac v1.1.1 integrated with QIIME 2 2024.5 [30], weighted UniFrac [31], Bray–Curtis dissimilarity [32], Shannon entropy [33], Chao1, Simpson diversity index [34], Simpson dominance index [34], and Gini index; and alpha correlation tests, beta correlation testing, alpha group significance testing, beta group significance testing, and multivariate analysis of variance with the “adonis” action. The principal coordinate analysis results, as well as the Bray–Curtis, weighted UniFrac, and unweighted UniFrac distance matrices and their transformations to UMAP and t-SNE, were plotted with use of the plugin “emperor” [35,36] for thorough examination of patterns in the data.

The main confounding factor identified was the sex of the patient, and the “adonis” analysis was run in two configurations: one without taking sex info formula as an interaction factor and the other with sex taken into account for permutations of variables tested.

Beta-diversity was assessed using Bray–Curtis dissimilarity and phylogenetic UniFrac-based distance matrices. Associations between microbial community structure and host-related variables were evaluated using PERMANOVA with 999 permutations. In the first step, univariable PERMANOVA models were used to evaluate individual demographic, clinical, inflammatory, dialysis-related, and body composition variables. Sex was treated both as a biologically relevant host variable and as a potential confounder because it was associated with microbiota diversity in the exploratory analyses. Therefore, where sample size permitted, additional models included sex as an adjustment factor or were repeated after stratification by sex. Vascular access type was evaluated as a dialysis-related clinical variable given its potential association with inflammation and dialysis-related exposures. However, analyses involving vascular access were considered exploratory due to an imbalance between access categories and further reduction in group sizes after sex stratification. The PERMANOVA results were therefore interpreted as evidence of differences in community structure only when the tested clinical groups were clearly defined and adequately represented. Because differences may influence PERMANOVA in within-group dispersion, homogeneity of multivariate dispersion was assessed for categorical variables using a distance-to-centroid approach where group sizes permitted. The PERMANOVA findings were interpreted cautiously when dispersion differed between groups or when subgroup sizes were small or imbalanced.

Because hemodialysis patients represent a clinically heterogeneous population, comorbidities, medication use, and dialysis-related exposures were considered potential confounders of microbiome–host associations. Available clinical covariates included demographic variables, selected comorbidities, medication use, dialysis-related characteristics, vascular access type, inflammatory markers, nutritional parameters, and body composition indices. Given the limited sample size and the large number of potential covariates, we did not construct fully saturated multivariable models including all available comorbidities and medication classes. Instead, we performed exploratory multivariable PERMANOVA/adonis analyses for beta-diversity metrics using selected clinically relevant covariates. These models included combinations of sex, vascular access type, body composition categories, and selected inflammatory or nutritional markers depending on data availability and model stability. The results of these analyses were interpreted as sensitivity analyses rather than definitive estimates of independent effects.

### 2.16. Stratification by Sex

The dataset was stratified by sex, and the entire diversity analysis was recalculated for both cohorts. The type of vascular access was detected as an interaction factor for the male cohort, and thus, two-way configuration of significance testing was also applied. The vascular access type class imbalance for the female cohort made the two-way configuration of significance testing impossible to apply.

### 2.17. Taxonomic Classification

Taxonomic classification was performed using pre-trained SILVA 138.2 human-stool-weighted scikit-learn classifier using sample sequences clustered to the SILVA 138.2 reference. Backwards compatibility between Qiime2 2026.1 and Qiime2 2025.7 allowed use of the pre-trained classifier. Krona plots were generated with stratification by sex; the q2-krona community plugin, utilizing the original krona library, was employed [37].

### 2.18. Missing Data Handling

Missing data were handled using a complete-case approach for each specific analysis. Because different analyses required different combinations of microbiome features, diversity metrics, clinical metadata, inflammatory markers, dialysis-related variables, and body composition parameters, the number of included participants varied between analyses. Alpha-diversity analyses were performed using samples with available diversity metrics and the corresponding clinical variable of interest. Beta-diversity and PERMANOVA analyses were performed using samples present in the relevant distance matrix and with complete metadata for the tested grouping variable. Analyses requiring matched microbiota and metadata distance matrices, including the Mantel test, were restricted to samples with complete overlap between both matrices.

## 3. Results

### 3.1. Patient Characteristics

The analysis included patient data such as age, sex, duration of hemodialysis in months, type of vascular access (catheter or arteriovenous fistula), dialysis adequacy expressed as the KT/V index, cause of kidney failure, and comorbidities, including cardiovascular disease, malignancy, diabetes, COPD, and autoimmune diseases, as well as medications used. Standard laboratory parameters were also included in the analysis, such as complete blood count, ferritin, transferrin, albumin, calcium, phosphorus, and parathyroid hormone (PTH) levels.

In our study population, no cases of ESA resistance were identified according to the KDIGO definition; that is, an EPO dose greater than 300 U/kg/week subcutaneously or 450 U/kg/week intravenously without an adequate hemoglobin response. Therefore, we considered the erythropoietin resistance index (ERI) to be a better indicator of ESA response, as it incorporates the mean hemoglobin level as one of its components. As in other studies on EPO hyporesponsiveness, we compared patient groups on either side of the median and/or across tertiles, since no universally accepted ERI cutoff clearly defines EPO hyporesponsiveness. In this study, the erythropoietin resistance index was calculated as the mean weekly erythropoietin dose per kilogram of body weight divided by the mean hemoglobin concentration (g/dL), based on values recorded over the previous three months.

The study included 96 hemodialysis patients, of whom 62 were men (64.6%) and 34 were women (35.4%). The median age was 66 years, and the median dialysis vintage was 25.5 months, indicating a population of predominantly older patients with established end-stage kidney disease. According to BMI-based classification, most participants had normal body weight (44.8%), whereas 32.3% were overweight and 22.9% were obese; no underweight individuals were identified.

Body composition analysis showed a median fat mass index of 8.2 kg/m^2^ and a median fat-free mass index of 19.3 kg/m^2^. The median skeletal muscle mass was 25.1 kg, visceral adipose tissue volume was 2.3 L, phase angle was 4.5, and total body water content was 52.7%. Altogether, these findings suggest considerable heterogeneity in nutritional and body composition status within the study group.

The median erythropoietin resistance index was 9.27 IU/kg/g/dL/week. Inflammatory markers showed a median IL-6 concentration of 6.92 pg/mL, hsCRP of 4.5 mg/L, IL-1β of 0.04 pg/mL, and TNF-α of 2.77 pg/mL, indicating the presence of low-grade systemic inflammation in a substantial proportion of patients. The median albumin concentration was 40 g/L, and the median transferrin concentration was 1.76 g/L. Dialysis adequacy was acceptable, with a mean Kt/V of 1.29. The median total MIS was 5, reflecting a generally mild to moderate burden of malnutrition–inflammation risk across the cohort.

### 3.2. Endpoint: Gut Microbiota Composition in HD Patients

The number of samples included differed between analyses because of incomplete overlap between microbiome data, clinical metadata, inflammatory markers, dialysis-related variables, and body composition measurements. Therefore, analyses were performed using complete cases for the variables required in each specific comparison. Alpha-diversity analyses included samples with available diversity metrics and matched clinical or body composition variables. PERMANOVA analyses included samples present in the relevant beta-diversity distance matrix and with complete metadata for the tested variable. The Mantel test required complete overlap between microbiota and metadata distance matrices, and was therefore restricted to 21 shared samples after exclusion of unmatched IDs.

**Figure 2:**
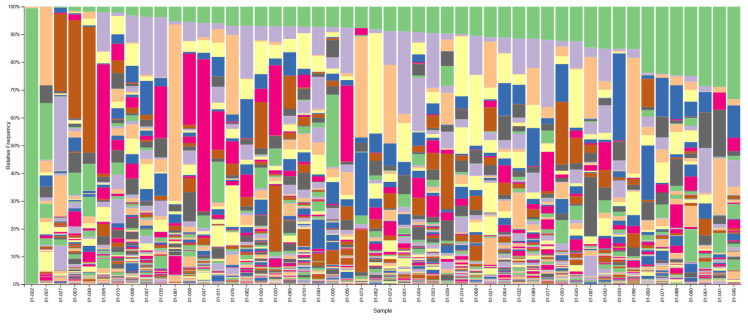
Stacked bar plot showing the relative abundance of taxa at the genus taxonomic level across individual samples. Each bar represents a single sample, and the total height of each bar corresponds to 100% of the microbial community composition. Different colors indicate different taxa, and their proportions reflect relative abundance. The plot illustrates variation in taxonomic composition among samples.

The relative abundance profiles at the genus taxonomic rank revealed marked inter-individual variability in microbial community composition. The communities were primarily composed of taxa belonging to the genera *Blautia*, *Faecalibacterium*, *Streptococcus*, *Gemmiger*, *Ruminococcus*, *Escherichia–Shigella*, and *Enterococcus*. While some samples were dominated by taxa commonly associated with the core gut microbiota, others showed an increased contribution of potentially opportunistic genera, including *Escherichia–Shigella*, *Streptococcus*, and *Enterococcus*, indicating substantial compositional heterogeneity across the cohort (Figure 2).

### 3.3. Secondary Endpoint: Sex-Associated Differences in Microbiota, Impact of Age and Visceral Adiposity on Diversity, Vascular Access and Inflammatory Markers

**Figure 3:**
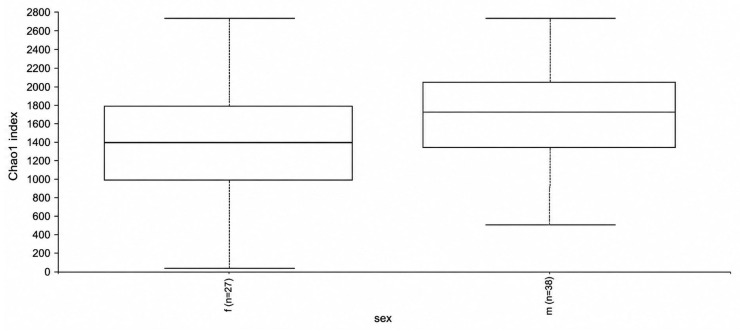
Alpha diversity (Chao1 index) across sex and selected body composition categories. A significant difference was observed between males (M) and females (K), with higher richness in men, whereas no significant differences were found across phase angle, VAT, FFMI, FMI, or BMI categories. Statistical comparisons were performed using the Kruskal–Wallis test.

Among the analyzed clinical and body composition variables, only sex was significantly associated with alpha diversity measured by the Chao1 index, with higher richness observed in men than in women (Figure 3, H = 4.38, *p* = 0.036). No significant differences were detected for phase angle, VAT class, FFMI class, FMI class, or BMI class (all *p* > 0.05), suggesting that microbial richness was relatively stable across the examined nutritional and body composition strata.

Beta-diversity analysis suggested a modest sex-associated difference in the gut microbial community structure. PERMANOVA indicated that community composition differed between women and men based on Bray–Curtis and phylogenetic UniFrac-based distance matrices (pseudo-F = 1.4658, *p* = 0.014, 999 permutations; n = 54). This result should be interpreted as evidence of a statistically detectable difference in multivariate community structure, rather than complete separation of the two groups.

In the alpha-diversity analyses, several significant associations with age and visceral adipose tissue (VAT) were observed. Shannon entropy showed positive correlations with both age (Spearman’s rho = 0.3626, *p* = 0.0071, n = 54) and scaled VAT values (rho = 0.3151, *p* = 0.0203, n = 54), indicating higher diversity in older participants and in individuals with greater visceral adiposity. Similarly, Chao1 richness was positively correlated with age (rho = 0.2946, *p* = 0.0172, n = 65) and scaled VAT (rho = 0.2779, *p* = 0.0250, n = 65). Faith’s phylogenetic diversity also increased with age (rho = 0.3173, *p* = 0.0194, n = 54).

In contrast, the Gini index showed negative correlations with both age (rho = −0.3402, *p* = 0.0056, n = 65) and scaled VAT (rho = −0.2881, *p* = 0.0199, n = 65), suggesting reduced community inequality with increasing age and visceral adiposity. Overall, these results indicate that both age and VAT were associated with greater microbial richness and diversity, accompanied by a more even community structure.

In the female subgroup, exploratory beta-diversity analysis was performed across normal-, elevated-, and high-VAT categories. PERMANOVA suggested differences in community structure across these categories (pseudo-F = 1.3416, *p* = 0.010, 999 permutations). However, because this analysis was restricted to women and involved relatively small subgroup sizes, the result should be regarded as exploratory. Ordination and distance-to-centroid plots showed partial overlap between categories, with the elevated-VAT group appearing most distinct.

**Figure 4:**
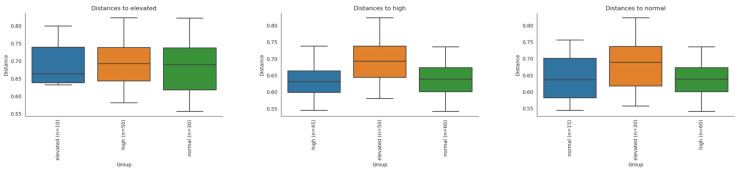
Beta-diversity differences among women stratified into the “normal”, “high”, and “elevated” categories. Overall group separation was assessed using PERMANOVA with 999 permutations. Boxplots show distances to group centroids, illustrating differences in microbial community composition between female subgroups.

Inspection of the group significance plots suggested partial separation of the female subgroups in multivariate space. The “elevated” category appeared to be the most distinct, whereas the “high” and “normal” groups showed greater overlap in their distance distributions. Overall, these findings support the presence of significant, although moderate, compositional differences in the gut microbiota among women across the analyzed categories (Figure 4).

To address the potential contribution of dialysis vascular access type to the relationship between systemic inflammation and gut microbiota composition, we constructed exploratory multivariable PERMANOVA models, including vascular access class, inflammatory markers, and selected body composition covariates. Across the tested beta-diversity metrics, vascular access type alone was not consistently associated with the gut microbiota composition. The lowest nominal association for vascular access class was observed in Bray–Curtis-based models but did not reach statistical significance after correction for multiple testing.

Exploratory interaction models suggested weak nominal associations involving vascular access class, body composition indices, and inflammatory markers, particularly absolute neutrophil count, NLR, TNF-α, ferritin, galectin-3, and GDF-15. The strongest unadjusted signals were observed for interactions involving vascular access class, fat mass index, or fat-free mass index and for absolute neutrophil count using UniFrac-based distances. However, these associations were small in effect size and did not remain significant after false discovery rate correction. Therefore, these results should be interpreted as hypothesis-generating rather than confirmatory. To evaluate whether dialysis vascular access type modified the association between systemic inflammation and gut microbiota composition, we performed exploratory PERMANOVA/adonis models, including vascular access class and selected inflammatory markers. Across Bray–Curtis, weighted UniFrac, and unweighted UniFrac distance matrices, vascular access type was not a robust independent determinant of gut microbiota beta-diversity. Nominal interaction signals were observed in selected models involving vascular access class, inflammatory markers and body composition variables, particularly absolute neutrophil count and NLR; however, none of these associations remained significant after correction for multiple testing. These findings suggest that vascular access type is unlikely to be a major independent driver of gut microbiota variation in this cohort, although subtle context-dependent inflammation-related interactions cannot be excluded.

**Figure 5:**
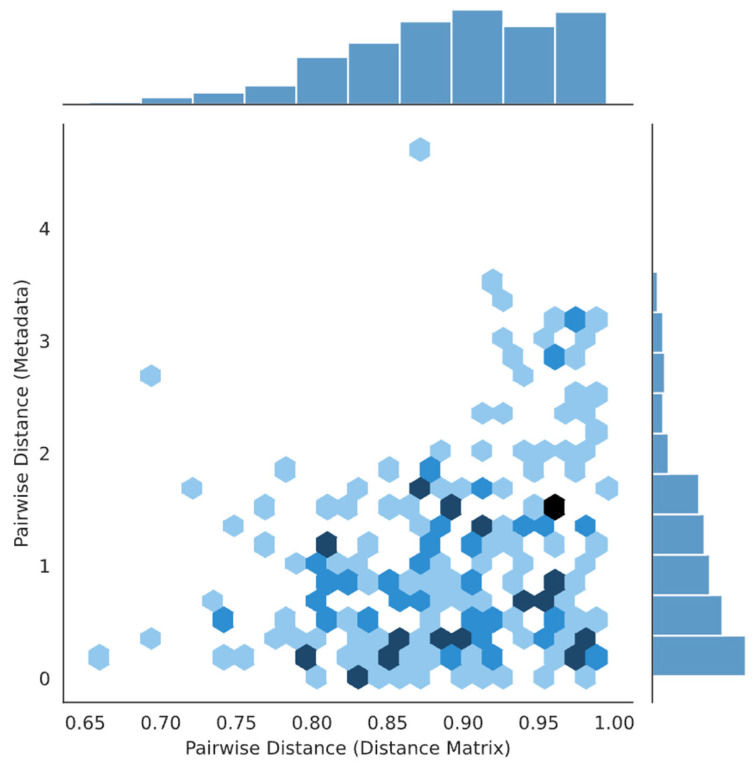
Mantel test assessing the association between the microbiota distance matrix and the metadata distance matrix. A positive correlation was observed between the two matrices (Spearman’s rho = 0.2414, *p* = 0.038; 999 permutations), indicating that larger differences in metadata were associated with greater differences in microbial community composition. The analysis was performed on 21 samples shared between both matrices. The color intensity reflects the number of observations within each bin: lighter blue hexagons indicate fewer pairwise comparisons, darker blue hexagons indicate higher local density, and black hexagons represent the highest-density bins.

As an exploratory analysis, a Mantel test was used to assess whether overall microbiota dissimilarity was associated with overall clinical metadata dissimilarity. The analysis was restricted to 21 samples with complete overlap between the microbiota and metadata distance matrices. A modest positive correlation was observed between the two matrices (Spearman’s rho = 0.2414, *p* = 0.038; 999 permutations). Given the reduced sample size, this result should be interpreted as supportive rather than definitive evidence that broader clinical dissimilarity may be reflected in the gut microbiota composition (Figure 5).

## 4. Discussion

In this study, we characterized the gut microbiota of maintenance hemodialysis patients in relation to demographic, nutritional, inflammatory, and body composition parameters. Several findings deserve emphasis. First, the microbiota showed marked inter-individual heterogeneity at the genus level, with profiles ranging from communities enriched in taxa commonly regarded as part of the core gut microbiota to profiles with a greater contribution of potentially opportunistic genera. Second, sex was associated with microbial richness, with higher Chao1 values in men than in women, and overall community composition also differed between the two sex groups. Third, age and visceral adipose tissue (VAT) were positively associated with several alpha-diversity metrics, whereas the Gini index showed inverse associations, suggesting a more even microbial community structure in older individuals and in those with greater visceral adiposity. Fourth, in women, beta-diversity differed across the analyzed categories, indicating possible sex-specific host–microbiota relationships. Finally, the positive Mantel test suggested that broader clinical dissimilarity between patients was modestly reflected in microbiota dissimilarity.

These observations fit the broader concept of the gut–kidney axis, in which chronic kidney disease and kidney failure are linked to altered intestinal ecology, impaired barrier function, systemic inflammation, and the accumulation of gut-derived uremic solutes. Prior work in kidney disease has shown that CKD itself alters the intestinal microbial structure, while more recent nephrology reviews have emphasized that uremia, inflammation, medication exposure, and dietary restriction all contribute to microbiome variation in CKD and dialysis populations. Accordingly, microbiome patterns in hemodialysis should probably be interpreted less as a simple healthy-versus-unhealthy dichotomy and more as a dynamic ecological response to the combined pressures of kidney failure, dialysis treatment, and host phenotype [4,38,39,40].

The marked between-patient heterogeneity observed in our cohort is also biologically plausible in light of prior dialysis-specific data. In a comparative study of patients undergoing hemodialysis or peritoneal dialysis, Stadlbauer et al. reported an increase in potentially pathogenic species and a decrease in beneficial species, with compositional changes in hemodialysis additionally associating with C-reactive protein. That framework is consistent with our finding that some samples were dominated by genera such as *Blautia* or *Faecalibacterium*, whereas others showed a greater contribution of *Escherichia–Shigella*, *Streptococcus*, or *Enterococcus*. Importantly, because our data are based on genus-level resolution, these taxa should not be overinterpreted functionally; rather, they support the view that the hemodialysis microbiome is compositionally unstable and clinically heterogeneous [40,41].

The sex-associated findings are particularly interesting. Men showed higher Chao1 richness than women, and beta-diversity analyses suggested overall compositional separation between the two groups. Recent large-cohort microbiome work has shown that sex-related differences in the gut microbiota are generally modest and are often strongly influenced by covariates, including age, menopause, and other host factors. Even so, these differences may still be relevant in clinical populations, especially when sex is linked to differences in body composition, fat distribution, immune tone, and treatment exposure. In the present cohort, the combination of alpha- and beta-diversity findings suggests that sex should not merely be treated as a nuisance covariate but, rather, as a biologically relevant stratifier in future dialysis microbiome studies [40,41,42].

The positive association between age and alpha-diversity metrics requires careful interpretation. In the aging literature, the gut microbiome is recognized as an important modulator of health across the lifespan, but age-related microbiome changes are not uniform and are influenced by diet, medications, physiology, and broader lifestyle-related factors. Moreover, recent work in healthy adults has shown that gut physiology, including transit time and luminal pH, is a major determinant of inter-individual microbiome variation. In this context, the higher Shannon, Chao1, and Faith’s phylogenetic diversity observed in older patients in our cohort should not be interpreted automatically as a marker of a “healthier” microbiome. In a dialysis setting, higher diversity may just as plausibly reflect ecological restructuring under chronic disease conditions, including the coexistence of commensal and opportunistic taxa. Thus, the age-related signal identified here is probably best understood as a marker of altered microbiome organization rather than a direct indicator of microbiological benefit [41,43,44].

A similarly cautious interpretation is needed for the associations with visceral adipose tissue. In our study, higher VAT was associated with greater richness and diversity and with lower Gini index values, suggesting a more even distribution of taxa. At first glance, this may appear counterintuitive because obesity is often discussed in the context of microbiome disruption. However, recent large-scale human data indicate that the gut microbiome’s composition and function are associated with multiple metabolic health measures, while experimental work has shown that obesity-associated microbiomes can be linked specifically to visceral adipose tissue inflammation. These observations suggest that adiposity-related microbiome shifts are not adequately captured by simplistic “high diversity good, low diversity bad” narratives. In hemodialysis patients, VAT may reflect not only metabolic burden but also nutritional reserve, inflammatory activity, and broader body composition phenotype. Therefore, our VAT-related findings likely point to a host–microbiota–metabolic interaction that is real but clinically complex [45,46].

The female subgroup analysis strengthens the possibility that host–microbiota relationships differ by sex. Among women, beta-diversity differed across the analyzed categories, with the “elevated” subgroup appearing the most distinct. Given the growing recognition that sex-stratified microbiome patterns can be subtle but meaningful, this result is worth retaining in the manuscript. At the same time, it should be framed as exploratory, particularly because subgroup numbers were limited and the biological interpretation depends heavily on the exact clinical variable used to define the “normal,” “high,” and “elevated” categories. The main value of this analysis is therefore not that it proves a sex-specific mechanism, but that it supports sex-aware hypothesis generation for future studies [42,46]. The PERMANOVA findings should be interpreted cautiously. Although sex and selected body composition categories were associated with beta-diversity, the corresponding ordination plots showed partial overlap between groups, indicating substantial inter-individual heterogeneity. Moreover, PERMANOVA may reflect differences in group centroids, differences in within-group dispersion, or both. Therefore, these results should not be interpreted as complete microbial separation between clinical categories but, rather, as evidence that selected host-related variables explain a modest proportion of variation in community composition.

The Mantel test was included as an exploratory, complementary analysis to examine whether global clinical dissimilarity was associated with global microbiota dissimilarity. Although a modest positive correlation was observed between the metadata and microbiota distance matrices, this finding should be interpreted cautiously because only 21 samples had complete overlap between both matrices. Thus, the result does not provide definitive evidence of multidimensional host–microbiota coupling but, rather, suggests a possible pattern in which broader clinical differences may be accompanied by differences in microbiota composition. This interpretation is consistent with the broader view that gut microbiota individuality reflects multiple host-, environment-, and phenotype-related factors rather than any single clinical variable alone [45,46].

Dietary intake represents a primary modulator of gut microbiota composition and function, particularly in the context of chronic kidney disease. In maintenance hemodialysis (HD) patients, the clinical necessity of restrictive diets—often low in fruits, vegetables, and whole grains to prevent hyperkalemia—results in a significant reduction in dietary fiber intake. This deficiency suppresses saccharolytic fermentation, leading to a decreased production of beneficial short-chain fatty acids (SCFAs), such as butyrate, which are essential for maintaining intestinal barrier integrity and exerting anti-inflammatory effects [4,16]. Conversely, the requirement for high protein intake to counteract protein–energy wasting (PEW) promotes an environment conducive to proteolytic fermentation. This metabolic shift increases the abundance of taxa capable of generating microbiota-derived uremic toxins, such as indoxyl sulfate and p-cresyl sulfate, which directly contribute to systemic inflammation and increased cardiovascular risk [3,18,40]. Given these associations, the role of different clinical diets in modulating the microbiota offers a promising therapeutic avenue. Interventions such as plant-dominant diets adapted for renal safety or targeted prebiotic and fiber supplementation could potentially restore microbial equilibrium and reduce the toxic metabolic burden [17,38]. Therefore, future longitudinal studies are warranted to investigate how standardized dietary interventions, tailored to the specific metabolic needs of the HD population, can selectively change the microbiota composition to improve host health and clinical outcomes.

Several limitations should be acknowledged. First, the cross-sectional design prevents causal inference. Second, the study was based on a single dialysis cohort without a healthy control group or a non-dialysis CKD comparator. Third, the analysis was performed at the genus level, which limits mechanistic interpretation. Fourth, some associations were detected in relatively small subgroups, and several findings should be regarded as exploratory, especially in the absence of a clearly reported multiple-testing correction strategy. Fifth, the Mantel analysis was based on a reduced set of shared samples. Finally, although ERI was an important clinical phenotyping variable in this study, the microbiome signals identified here aligned more clearly with sex, age, and body composition parameters than with ESA responsiveness per se.

Despite these limitations, the study has several strengths. The cohort was clinically well characterized, with simultaneous assessment of inflammatory markers, nutritional indicators, dialysis adequacy, body composition, and ERI. This multidimensional phenotyping is important because the current nephrology and microbiome literature increasingly argues that CKD-associated dysbiosis should be analyzed in the context of host metabolic and inflammatory state, rather than as an isolated sequencing readout. In addition, the inclusion of bioimpedance-derived markers, such as VAT, phase angle, FMI, and FFMI, adds clinically meaningful granularity beyond BMI alone [38,45,47].

## 5. Conclusions

In conclusion, our results suggest that the gut microbiota of hemodialysis patients is highly heterogeneous and associated primarily with sex, age, visceral adiposity, and broader host phenotype. These associations are statistically modest but biologically plausible, and support the view that microbiome variation in dialysis reflects the combined influence of demographic, physiological, metabolic, and treatment-related factors. Future studies should validate these findings in larger, longitudinal, and sex-stratified cohorts; incorporate dietary and medication data more explicitly; and extend the analysis toward species-level and functional profiling to better define the clinical relevance of microbiota alterations in end-stage kidney disease. Future studies should focus on the impact of specific clinical dietary interventions, such as fiber-enriched diets or plant-dominant low-protein diets (PLADO), on the gut microbiota composition in the HD population. Investigating how these nutritional strategies could reduce uremic toxin production through microbiota modulation remains a crucial step toward personalized kidney-protective therapy.

## 6. Strengths

The study group was assessed not only with a nutritional questionnaire but also with examination of body composition. Erythropoietin resistance was calculated over 3 months of treatment.

## 7. Limitations

This study has several limitations. First, it was a single-center, cross-sectional study, which limits causal interpretation and generalizability. Second, although short-term follow-up information was available, the number of outcome events was insufficient for robust prognostic modeling; therefore, clinical outcomes were not included in the main inferential framework. Third, some analyses were based on different sample sizes because of incomplete overlap between microbiome data, clinical metadata, inflammatory markers, dialysis-related variables, and body composition measurements. Fourth, dietary intake, socioeconomic status, and detailed medication exposure were not systematically captured, although these factors may influence gut microbiota composition. Finally, the use of a closed-reference 97% OTU approach provided a conservative reference-anchored profile but may have excluded novel or poorly represented taxa.

## Figures and Tables

**Table 1:** Group characteristics.

Variable	Overall Participants	Women	Men	*p*-Value
Overall participants	*n* = 96	*n* = 34	*n* = 62	
Age [years]	Median: 66; IQR = 51.0–73.3	Median: 70; IQR = 51.2–73.0	Median: 66; IQR = 49.5–74.0	1.000
Dialysis vintage [months]	Median: 25.5; IQR = 14.0–46.3	Median: 20.0; IQR = 14.0–29.5	Median: 29.5; IQR = 14.0–54.8	0.056
Patients’ nutrition by BMI [%]	Underweight: n = 0 (0.0%); Normal weight: n = 43 (44.8%); Overweight: n = 31 (32.3%); Obese: n = 22 (22.9%)	Underweight: n = 0 (0.0%); Normal weight: n = 12 (35.3%); Overweight: n = 11 (32.4%); Obese: n = 11 (32.4%)	Underweight: n = 0 (0.0%); Normal weight: n = 31 (50.0%); Overweight: n = 20 (32.3%); Obese: n = 11 (17.7%)	0.212
FMI [kg/m^2^]	Median: 8.2; IQR = 4.0–11.5	Median: 10.3; IQR = 6.7–14.9	Median: 6.2; IQR = 2.8–9.1	<0.001
FFMI [kg/m^2^]	Median: 19.3; IQR = 17.2–21.4	Median: 17.5; IQR = 16.1–19.7	Median: 19.9; IQR = 18.3–22.4	<0.001
SMM [kg]	Median: 25.1; IQR = 19.9–29.8	Median: 19.9; IQR = 16.5–25.3	Median: 26.6; IQR = 24.1–31.8	<0.001
VAT [L]	Median: 2.3; IQR = 0.9–4.4	Median: 1.6; IQR = 0.9–3.6	Median: 2.7; IQR = 1.2–4.5	0.052
PhA	Median: 4.5; IQR = 3.9–5.5	Median: 4.5; IQR = 3.8–5.4	Median: 4.6; IQR = 3.9–5.5	0.763
TBW [%]	Median: 52.7; IQR = 46.0–61.1	Median: 45.8; IQR = 41.7–52.9	Median: 55.8; IQR = 49.1–65.1	<0.001
ERI [IU/kg/g/dL/week]	Median: 9.27; IQR = 4.14–18.68	Median: 9.61; IQR = 5.21–21.34	Median: 9.19; IQR = 2.87–17.77	0.258
IL-6 [pg/mL]	Median: 6.92; IQR = 3.90–12.29	Median: 6.60; IQR = 4.81–11.45	Median: 7.77; IQR = 3.04–13.03	0.988
hsCRP [mg/L]	Median: 4.5; IQR = 2.3–17.3	Median: 4.8; IQR = 2.6–17.7	Median: 4.1; IQR = 2.2–17.0	0.646
IL-1β [pg/mL]	Median: 0.04; IQR = 0.00–0.16	Median: 0.05; IQR = 0.00–0.14	Median: 0.01; IQR = 0.00–0.16	0.423
TNF-α [pg/mL]	Median: 2.77; IQR = 2.24–3.65	Median: 2.72; IQR = 2.17–3.35	Median: 2.84; IQR = 2.29–4.07	0.344
Albumin [g/L]	Median: 40; IQR = 37.0–41.0	Median: 40; IQR = 37.2–41.0	Median: 40; IQR = 37.0–41.0	0.917
Transferrin [g/L]	Median: 1.76; IQR = 1.55–2.02	Median: 1.78; IQR = 1.48–1.95	Median: 1.75; IQR = 1.59–2.03	0.797
Kt/V	Mean: 1.29 ± 0.25	Mean: 1.41 ± 0.27	Mean: 1.23 ± 0.21	<0.001
Total MIS	Median: 5; IQR = 4.0–9.0	Median: 5; IQR = 4.0–7.8	Median: 5; IQR = 4.0–9.0	0.674

Abbreviations: IQR—interquartile range, BMI—body mass index, ERI—erythropoietin resistance index, MIS—malnutrition inflammation scale, Kt/V—dialysis adequacy index.

**Table 2:** Definitions and interpretation of body composition and anthropometric parameters used in the study.

Parameter	Description
BMI—body mass index [kg/m^2^]	A value derived from body mass divided by the square of the body height, traditionally used to group individuals as underweight, normal, overweight, or obese.
FFM—fat-free mass [kg], relative to weight [%]	Calculated by subtracting body fat weight from total body weight; also referred to as “lean body mass.”
FFMI—fat-free mass index [kg/m^2^]	Describes the amount of fat-free mass (“lean body mass”) in relation to height and weight. Similar to BMI.
FM—fat mass [kg], relative to weight [%]	Total amount of fat; percentage of total bodyweight that is fat.
FMI—fat mass index [kg/m^2^]	Describes the amount of fat mass in relation to height and weight. Similar to BMI.
TBW—total body water [l], relative to weight [%]	The sum of intracellular water and extracellular water volume; approx. 60% of body weight of a normovolemic individual.
Phase angle φ [°]	Calculated by reactance/resistance ratio during bioelectrical impedance measurement. Used as an indicator of cell wall stability. Helpful in health risk assessment.
VAT—visceral adipose tissue [l]	Also known as abdominal fat, describes adipose tissue that surrounds the organs in the abdominal cavity. Overdeposition of visceral fat in the abdomen is known as visceral obesity.

## Data Availability

For additional data, please contact ewa.kwiatkowska@pum.edu.pl.

## Abbreviations

The following abbreviations are used in this manuscript:

16S rRNA	16S ribosomal RNA
BAM	Binary Alignment/Map
BMI	body mass index
CKD	chronic kidney disease
COPD	chronic obstructive pulmonary disease
CRP	C-reactive protein
EDTA	ethylenediaminetetraacetic acid
ELISA	enzyme-linked immunosorbent assay
EPO	erythropoietin
ERI	erythropoietin resistance index
ESA	erythropoiesis-stimulating agent
ESRD	end-stage renal disease
FASTA	FAST-All sequence format
FFM	fat-free mass
FFMI	fat-free mass index
FM	fat mass
FMI	fat mass index
HAC	high accuracy
HD	hemodialysis
HRT	hydraulic retention time
hsCRP	high-sensitivity C-reactive protein
IL-1β	interleukin-1 beta
IL-6	interleukin-6
IQR	interquartile range
KDIGO	Kidney Disease: Improving Global Outcomes
Kt/V	dialysis adequacy index
MIA	malnutrition–inflammation–atherosclerosis
MIS	malnutrition–inflammation score
ONT	Oxford Nanopore Technologies
OTU	operational taxonomic unit
PEW	protein–energy wasting
PhA	phase angle
PTH	parathyroid hormone
qPCR	quantitative polymerase chain reaction
SCFA	short-chain fatty acid
SMM	skeletal muscle mass
SUP	super accuracy
TBW	total body water
TMAO	trimethylamine *N*-oxide
TNF-α	tumor necrosis factor alpha
TSV	tab-separated values
UMAP	uniform manifold approximation and projection
VAT	visceral adipose tissue
